# *De novo* assembly of the pennycress (*Thlaspi arvense*) transcriptome provides tools for the development of a winter cover crop and biodiesel feedstock

**DOI:** 10.1111/tpj.12267

**Published:** 2013-06-20

**Authors:** Kevin M Dorn, Johnathon D Fankhauser, Donald L Wyse, M David Marks

**Affiliations:** 1 Department of Plant Biology, University of Minnesota1445 Gortner Avenue, 250 Biological Sciences Center, Saint Paul, MN, 55108, USA; 2 Department of Agronomy and Plant Genetics, University of Minnesota411 Borlaug Hall, 1991 Upper Buford Circle, Saint Paul, MN, 55108, USA

**Keywords:** *Thlaspi arvense*, pennycress, RNA–seq, *de novo* assembly, comparative transcriptomics, translational research

## Abstract

Field pennycress (*Thlaspi arvense* L.) has potential as an oilseed crop that may be grown during fall (autumn) and winter months in the Midwestern United States and harvested in the early spring as a biodiesel feedstock. There has been little agronomic improvement in pennycress through traditional breeding. Recent advances in genomic technologies allow for the development of genomic tools to enable rapid improvements to be made through genomic assisted breeding. Here we report an annotated transcriptome assembly for pennycress. RNA was isolated from representative plant tissues, and 203 million unique Illumina RNA-seq reads were produced and used in the transcriptome assembly. The draft transcriptome assembly consists of 33 873 contigs with a mean length of 1242 bp. A global comparison of homology between the pennycress and Arabidopsis transcriptomes, along with four other Brassicaceae species, revealed a high level of global sequence conservation within the family. The final assembly was functionally annotated, allowing for the identification of putative genes controlling important agronomic traits such as flowering and glucosinolate metabolism. Identification of these genes leads to testable hypotheses concerning their conserved function and to rational strategies to improve agronomic properties in pennycress. Future work to characterize isoform variation between diverse pennycress lines and develop a draft genome sequence for pennycress will further direct trait improvement.

## Introduction

Plant-derived biofuels have the potential to reduce carbon emissions and provide a renewable source of energy (Hill *et al*., 2006). Replacement of fossil fuels with those derived from plant biomass or oilseeds holds promise to slow global climate change due to anthropological release of greenhouse gasses. The increased access and affordability of next-generation sequencing resources (i.e. genomics and transcriptomics) provides new approaches for rapidly employing new plant species for use as biofuel feedstock. Many plant species are being considered not only as new sources of biofuel, but also as components of the landscape that improve the environment. Species that have only been recently removed from the wild will need to be modified in ways that remove their weedy traits while enhancing their agronomic properties. Application of next-generation sequencing resources in the development of candidate species should allow rapid advancement and improvement in these species (Varshney *et al*., 2009).

Biofuel crop species that do not displace land for food production or encourage the destruction of natural lands are especially attractive as alternatives to the biofuel standard: corn-derived ethanol (Fargione *et al*., 2008; Tilman *et al*., 2009). In addition, new species that provide ecosystem services to reduce the effects of large-scale intensive farming are essential to ensure food security. This is especially important in the Midwestern United States, where large portions of the land dedicated to agriculture are left barren for almost half the year, from the time of harvest until the next crop establishment. Planting winter annual crops following the fall harvest has been shown to alleviate soil degradation, topsoil loss through erosion and nutrient run-off, to help prevent water pollution by scavenging excess nitrogen from the soil, and to limit spring weed growth (Dabney *et al*., 2001; Snapp *et al*., 2005).

Pennycress is especially attractive because it provides a winter cover that uses excess nitrogen and slows soil erosion, provides a spring cover that suppresses weeds, and yields a harvestable oilseed. The combination of these traits makes pennycress one of the best candidate biofuel plant species. Pennycress may be harvested in the spring using conventional machinery, and yields up to 1345 kg seed/hectare (Best and Mcintyre, 1975; Mitich, 1996). Pennycress seeds are high in oils that can easily be converted into biodiesel (Moser *et al*., 2009a,b; Boateng *et al*., 2010; Isbell and Cermak, 2012; Hojilla-Evangelista *et al*., 2013). A recent study showed that pennycress may be planted as a winter cover crop after corn in the fall (autumn) and harvested in the spring without impeding subsequent soybean cultivation, or dramatically affecting soybean yield, protein content and oil quantity and quality (Phippen and Phippen, 2012). Thus, the use of pennycress does not require any new land or displace traditional food crops. A recent life cycle assessment indicated that pennycress-derived fuels could qualify as advanced biofuels under the US Environmental Protection Agency Renewable Fuels Standard (Fan *et al*., 2013).

While the inherent agronomic properties of pennycress are already good, efforts are required to maximize oilseed yield, content and composition while reducing seed dormancy and glucosinolate content. Previous studies compared various genetic aspects of pennycress with those of its close relative *Thlaspi caerulescens*, which hyper-accumulates zinc and cadmium (Hammond *et al*., 2006; Milner and Kochian, 2008). Analysis of over 600 pennycress ESTs revealed a close relationship between pennycress and Arabidopsis (Sharma *et al*., 2007). The limited genetic divergence between Arabidopsis and its wild relatives, such as pennycress, facilitates translation of basic knowledge gleaned from years of Arabidopsis research.

Here we report the sequencing, *de novo* assembly and annotation of the transcriptome of several pennycress tissues, including roots, leaves, shoots, flowers and seed pods. The draft transcriptome consists of 33 873 transcripts. Comparative analyzes versus other Brassicaceae species showed a high degree of conservation, which serves as a validation of the assembly. Comparative analysis versus *Arabidopsis thaliana* allowed us to identify many pennycress orthologs that are probably responsible for controlling flowering time and glucosinolate metabolism. This pennycress dataset, together with further development of genomic tools and germplasm resources will provide unprecedented tools for starting a breeding program.

## Results

### Generation of RNA-seq reads and *de novo* assembly

RNA was isolated from five pennycress tissue types and sequenced on a single land of the Illumina HiSeq 2000 platform (100 bp paired-end), yielding 374 725 460 reads with a mean quality score >Q30 (see Experimental Procedures). After removing duplicate reads, trimming adaptors, and filtering for low quality sequences, a total of 203 003 444 unique, clean reads were obtained, with a mean length of 87.6 bp. The full, unfiltered short-read dataset was deposited in the National Center for Biotechnology Information (NCBI) Short Read Archive under accession number SRR802670.

The filtered reads were *de novo* assembled using the CLC Genomics Workbench software package. The effect of varying *de novo* assembly parameters was examined by performing 41 separate assemblies. Word size (*k*-mer), match length (the percentage length of a read required to match the initial contig build), and the match percentage (the percentage sequence identity required to match a read to the initial contig build) were varied, and the effect on various assembly statistics was examined. Regardless of match length and percentage, assemblies with smaller word sizes had smaller mean contig lengths. Assemblies with smaller word sizes also assembled a few contigs that were significantly larger (16–18 kb) compared to assemblies with word sizes ≥52 (15 kb). These large contigs are probably mis-assembled because each contained sequences similar to multiple Arabidopsis genes. The assemblies created with 95% match length and 95% match percentage parameters were chosen for further comparison of how word size affected the relative assembly quality. Increasing the word size caused the percentage of reads used in the final assembly and the mean contig length to increase, while decreasing the number of contigs assembled. The assemblies with larger word sizes also had a higher percentage of contigs that had at least 1 BLASTX hit to at least one Arabidopsis peptide. The statistics regarding the assembly optimization and blast results for the assembly with word size 64, 95% match length and 95% match percentage are shown in Table S1.

**Figure 1:**
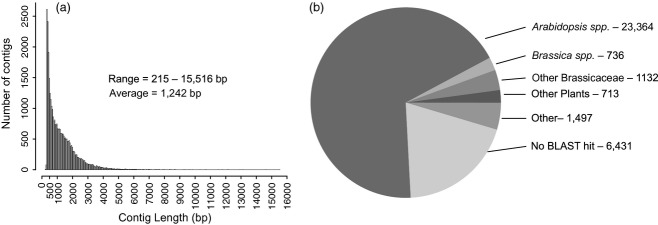
Contig length distribution and taxonomic distribution of top annotation hits. (a) Histogram of the length distribution of assembled contigs. (b) Taxonomic distribution of the top blast hits for each transcript in the *de novo* transcriptome assembly from Blast2GO. Only taxonomic data for the top blast result of each transcript are shown.

The assembly with a word size of 64, 95% match length, and 95% match percentage was chosen for further analysis and annotation due to the high quality of assembly statistics and high proportion of assembled transcripts with significant matches to Arabidopsis genes compared to the other assemblies. A summary of sequencing reads and assembly statistics is shown in Table 1. A total of 33 874 contigs were assembled using these parameters. This includes a spiked phiX174 genome sequence that serves as a sequencing control, which was subsequently removed from the final assembly and total assembly length. The mean contig length was 1242 bp, with minimum and maximum contig lengths of 215 and 15 516 bp, respectively. The size distribution of contig lengths is shown in Figure 1(a). The N50 was 1729 bp, meaning all contigs this size or larger encompassed 50% of the total 42 069 800 bp assembly length. This Transcriptome Shotgun Assembly project has been deposited at DDBJ/EMBL/GenBank under the accession GAKE00000000. The version described in this paper is the first version, GAKE01000000. Approximately 1.5% of the contigs were excluded from the archives due to the number of ambiguous nucleotides in those sequences. The complete, annotated FASTA file is available at http://www.cbs.umn.edu/lab/marks/pennycress/transcriptome.

**Table 1:** Illumina RNA-seq reads and *de novo* assembly statistics

Parameter	Value
Number of raw unfiltered reads	374 725 460
Total length of reads pre-filtering (bp)	37 472 546 000
Total length of reads post-filtering (bp)	17 799 652 172
Number of trimmed unique reads	203 003 444
Number of contigs	33 873
Mean contig length (bp)	1242
Minimum/maximum contig length (bp)	215/15 516
N50 (bp)	1729
Total assembly length (bp)	42 069 800

A pooled RNA sample consisting of representative plant tissues was sequenced using the Illumina HiSeq 2000 platform (2 × 100 bp). Duplicate reads were removed first, then filtered for quality score, trimmed, and assembled into contigs using the *de novo* assembly tool in CLC Genomics Workbench.

### Annotation and functional characterization of pennycress transcripts

The pennycress transcriptome sequences were annotated using Blast2GO Pro (Conesa *et al*., 2005). The database used in this analysis only contains well-characterized sequences and does not include sequences from resources such as newly assembled draft genomes. The taxonomic distribution from this analysis was examined (Figure 1b). Over 20 000 transcripts had top hits to an Arabidopsis species, including 11 936 transcripts with a top hit to *A. thaliana*, and 11 364 transcripts with a top hit to *Arabidopsis lyrata*. Almost 75% of the pennycress transcripts had top blast hits within the Brassicaceae family. Species of the sister genus, *Brassica*, had a large proportion of these top hits: *Brassica rapa* (283), *Brassica napus* (233) and *Brassica oleraceae* (164). Top matches to plant sequences outside the Brassicaceae were found for 713 transcripts. Overall, approximately 23% of the transcripts had top blast hits to either non-plant sequences or lacked significant similarity to any sequence in the public database (Figure 1b). The complete dataset from the final assembly including annotations and associated GO terms from this analysis is provided in Table S2.

Annotations and associated cellular component, molecular function and biological process gene ontology (GO) terms were produced for each pennycress transcript. A total of 27 456 transcripts had a significant hit in the public databases (blast *E*-value ≪ 0.01), and 26 797 transcripts received at least one GO annotation. The most highly represented biological process GO terms were oxidation/reduction processes (1403 transcripts) and DNA-dependent regulation of transcription (1255 transcripts). GO terms associated with response to cold (727 transcripts), the vegetative to reproductive phase transition of the meristem (462 transcripts) and the regulation of flower development (411) were also highly represented. The 50 most highly represented GO terms are shown in Figure S1.

### Comparative transcriptomics of pennycress versus other Brassicaceae species

**Figure 2:**
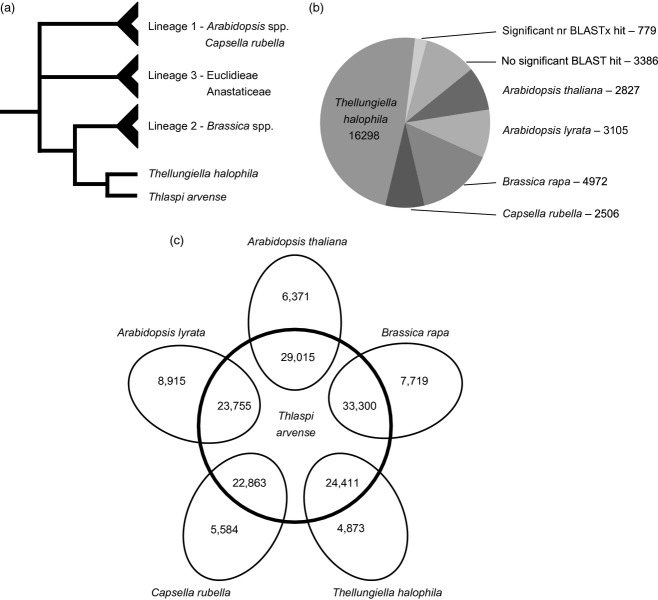
Comparative transcriptomics of pennycress versus five Brassicaceae species. (a) Representation of the Brassicaceae phylogeny, adapted from Beilstein *et al*. (2010) and Franzke *et al*. (2011). (b) BLASTx comparison of the pennycress transcriptome assembly versus *A. thaliana*, *Arabidopsis lyrata*, *Brassica rapa*, *Capsella rubella* and *Thellungiella halophila*. The top blast hit (*e* ≤ 0.05) for each pennycress transcript versus the five species is shown. Contigs without significant hits were then compared to the NCBI peptide non-redundant database. (c) Five pairwise tBLASTn comparisons of Brassicaceae species to the pennycress transcriptome assembly. Sequences with significant homology (*e* ≤ 0.05 and positive match percentage ≧70%) shared between the five Brassicaceae species and pennycress (*Thlaspi arvense*) are shown in the inner circle.

Previous molecular analyzes of the Brassicaceae have divided the family into three basic lineages, recently reviewed by Franzke *et al*. (2011). *Thlaspi arvense* is a member of expanded lineage 2, and is more closely related to *Thellungiella halophila* and other *Eutrema*/*Thellungiella* species than the *Brassica* species in lineage 2 (Figure 2a). *Arabidopsis thaliana*, *A. lyrata* and *Capsella rubella* are members of lineage 1. To explore the relationship between pennycress and other Brassicaceae at the transcriptome level, we compared the assembled translated pennycress transcriptome to a peptide database derived from the sequenced genomes of *A. thaliana*, *A. lyrata*, *C. rubella*, *B. rapa* and *T. halophila*. A BLASTx comparison of the pennycress transcriptome with this peptide database showed that 16 298 of the 33 873 pennycress contigs had significant (*e* ≤ 0.05) top hits to *T. halophila* (Figure 2b). *B. rapa* had the next highest number of top hits (4972), with the lineage 1 species having approximately 3000 top hits each. A BLASTx comparison of the remaining sequences without significant hits to one of the five Brassicaceae species revealed that 3386 sequences had no significant hit in the NCBI non-redundant peptide database. This blast search returned 779 pennycress contigs with significant hits in the non-redundant peptide database, including 424 fungi. Many of these fungal hits (273) were to fungal plant pathogens, including *Fusarium*, *Pyrenophora*, *Phaeosphaeria*, *Leptosphaeria* and *Bipolaris* species (Table S3). These fungal transcripts were left in the assembly as the association between pennycress and these fungi may be informative in future analyzes.

To examine the degree of conservation between pennycress and other sequenced Brassicaceae species, five pairwise tBLASTn comparisons were performed between pennycress and each of the five Brassicaceae species (Figure 2c). *Thellungiella halophila* had the highest number of sequences with significant hits to the pennycress database (*e* ≤ 0.05 and ≥70% positive match percentage), together with the greatest proportion of peptides with significant matches (24 411/29 284). All five species had at least 72% of their proteins significantly represented in the pennycress database. All five Brassicaceae genomes share 14 677 of the pennycress transcripts (*e* ≤ 0.05 and ≥70% positive match percentage). An additional 4547 sequences were shared between pennycress and at least one of the other Brassicaceae species. The tBLASTn results from this analysis are provided in Table S4. A global view of the top pennycress transcripts and the similarity to each *A. thaliana* peptide (primary transcripts only) is shown in Figure S2. Of the 27 416 Arabidopsis loci, 14 186 had transcripts with >70% similarity and >70% coverage in the pennycress transcriptome.

**Figure 3:**
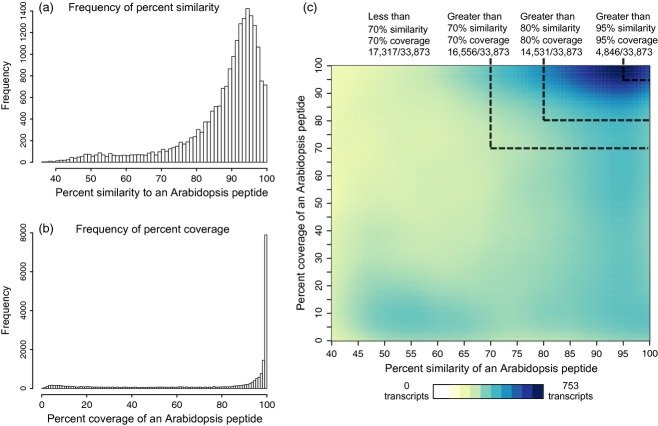
Similarity and coverage of pennycress transcripts versus Arabidopsis genes. (a) Histogram showing frequency versus percentage similarity (positive amino acid identity) of pennycress contigs versus an Arabidopsis peptide. (b) Histogram showing frequency versus percentage coverage (longest positive hit/peptide length) of pennycress contigs versus an Arabidopsis peptide. Most assembled pennycress transcripts have high coverage which greatly skews the histogram to the right. (c) Smoothed color density representation of the percentage similarity (*x* axis) of each pennycress transcript plotted against the percent coverage of the Arabidopsis protein similarity (*y* axis). The plot was produced using the ‘smoothScatter’ function in r (R Development Core Team, 2008), which produces a smoothed density representation of the scatterplot using a kernel density estimate (nbin = 100). Darker color indicates a higher density of transcripts in a given position, with the darkest ‘bin’ containing over 700 transcripts. Boxes encompassing transcripts encoding peptides with 70, 80 and 95% sequence similarity and coverage are shown in the upper right corner. Raw similarity and coverage data are provided in Table S2.

To more closely examine the level of global sequence conservation between pennycress and *A. thaliana*, we further examined a BLASTx comparison of the pennycress transcriptome assembly to the Arabidopsis TAIR10 peptide database (primary transcripts only). The relative homology of each predicted peptide to the most similar Arabidopsis protein was measured by the percentage of positive sequence similarity (Figure 3a) and percentage coverage (Figure 3b). A smooth scatter plot representing the percentage similarity and percentage coverage for each pennycress sequence compared to the closest Arabidopsis peptide sequence is shown in Figure 3(c). A large proportion (>85%) of transcripts show at least 70% similarity to an Arabidopsis protein. A total of 16 556 pennycress predicted peptides had at least one match to an Arabidopsis gene with >70% similarity/>70% coverage (Figure 3c, boxes), of which 4846 pennycress transcripts showed ≥95% similarity and coverage, 9685 transcripts showed between 80 and 95% similarity and coverage, and 2025 transcripts showed between 70 and 80% similarity and coverage. A total of 17 317 transcripts showed <70% similarity and coverage, and 4783 transcripts lacked a significant BLASTx hit (*e* ≤ 0.05) to an Arabidopsis peptide.

### Identification of candidate pennycress genes controlling flowering time and glucosinolate levels

The close evolutionary relationship between pennycress and Arabidopsis enabled identification of potential pennycress orthologs responsible for controlling important agronomic traits such as time to flower and glucosinolate metabolism. For each pennycress transcript, the top 20 BLASTx hit against the Arabidopsis peptide database was mined for hits to Arabidopsis genes known to control these traits. For these transcripts, the longest theoretical translation was obtained to explore protein sequence conservation. The nucleotide sequences and predicted peptides for each sequence, along with the amino acid alignment to their respective Arabidopsis homolog, are shown in Data S1.

To investigate the conservation of the flowering time pathway in pennycress, we attempted to reconstruct the flowering pathway in Arabidopsis using predicted peptides from the transcriptome assembly (Jung and Müller, 2009). Full-length predicted peptides (methionine to stop codon) with high homology to their respective Arabidopsis peptides were obtained for VERNALIZATION1 (*At3G18990*), VERNALIZATION2 (*At4G16845*), VERNALIZATION INSENSITIVE 3 (*At5G57380*), LIKE HETEROCHROMATIN PROTEIN 1 (*At5G17690*), FLOWERING LOCUS C (*At5G10140*), SHORT VEGETATIVE PHASE (*At2G22540*), TWIN SISTER OF FT (*At4G20370*), AGAMOUS-LIKE 19 (*At4G22950*), SUPPRESSOR OF OVEREXPRESSION OF CO 1 (*At2G45660*), FLOWERING LOCUS T (*At1G65480*), LATE ELONGATED HYPOCOTYL (*At1G01060*), TIMING OF CAB EXPRESSION 1 (*At5G61380*), PSEUDO-RESPONSE REGULATOR 7 (*At5G02810*), PSEUDO-RESPONSE REGULATOR 9 (*At2G46790*), FLAVIN-BINDING KELCH REPEAT F BOX 1 (*At1G68050*), and GIGANTEA (*At1G22770*). Partial or incomplete matches were found for MULTICOPY SUPRESSOR OF IRA1 (*At5G58230*), ATBZIP14/FD (*At4G35900*), TERMINAL FLOWER 1 (*At5G03840*), APETALA1 (*At1G69120*), FRUITFULL (*At5G60910*), CIRCADIAN CLOCK ASSOCIATED 1 (*At2G46830*), and CONSTANS (*At5G15840*). None of the pennycress transcripts had a top hit to the Arabidopsis FRIGIDA (FRI) locus (*At4G00650*); however, we found a 613 amino acid predicted peptide similar to *B. napus* FRI.a (GenBank accession number AFA43306.1), which was previously shown to be a major determinant of flowering in rapeseed (Wang *et al*., 2011a). No pennycress ortholog for LEAFY (*At5G61850*) was found in the final assembly. However, truncated transcripts similar to the Arabidopsis LEAFY sequence were detected in the assemblies created using word sizes of 24, 30 and 46 (95% match length and percentage). Putative orthologs of the FRIGIDA protein complex were also found (Choi *et al*., 2011). Full-length predicted peptides with high sequence similarity were found for EARLY FLOWERING IN SHORT DAYS (*At1G77300*), SUPPRESSOR OF FRIGIDA4 (*At1G30970*), FLC EXPRESSOR (*At2G30120*), FRIGIDA-ESSENTIAL 1 (*At2G33835*), FRIGIDA LIKE 1 (*At5G16320*), and HOMOLOG OF YEAST YAF9 A (*At5G45600*). No unique matches were found for TBP-ASSOCIATED FACTOR 14 (*At2G18000*). A reconstruction of the flowering time pathway using pennycress transcripts is shown in Figure S3.

Through comparative transcriptomics, we have identified potential orthologs of both myrosinases and specifier proteins responsible for controlling the breakdown of glucosinolates in pennycress. We performed a BLASTX comparison of the pennycress transcriptome assembly against the Arabidopsis proteome for the main myrosinases TGG1-6 (THIOGLUCOSIDE GLUCOHYDROLASE 1-6) and PEN2 (PENETRATION 2), and specifier proteins ESP (EPITHIOSPECIFIER PROTEIN) and NSP1-5 (NITRILE SPECIFIER PROTEIN 1-5) that are responsible for glucosinolate breakdown in Arabidopsis. An Arabidopsis TGG1 ortholog was found whose longest ORF produced a predicted peptide with high sequence conservation compared to Arabidopsis. The top BLASTp hit against the non-redundant protein database was a *Eutrema wasabi* myrosinase (GenBank accession number BAE16356). The predicted peptide from another pennycress transcript was found to be highly similar to Arabidopsis TGG4. This predicted peptide had a top BLASTp hit to a myrosinase from *Amoracia rusticana* (horseradish) (GenBank accession number AEZ01595.1). An ortholog for the Arabidopsis atypical myrosinase PEN2 was also found. The pennycress PEN2 predicted peptide has 95% sequence identity conservation to an unnamed protein product from *T. halophila* (GenBank accession number BAJ34425.1).

The conservation of specifier proteins was also examined. Three pennycress transcripts were found to have high homology to three Arabidopsis NSP genes. A full-length predicted peptide was similar to the AtNSP1 peptide, but was most similar to the nitrile-specifier protein from another member of the Brassicaceae, *Schouwia purpurea* (GenBank accession number AFP47629.1). Another transcript was found that encoded a 1073 amino acid predicted peptide with high sequence similarity to the C-terminal Kelch domain-containing region of AtNSP4. The N-terminus of this peptide has high similarity to other AtNSPs. A third transcript was found to encode a predicted peptide with high similarity to AtNSP5. We also identified orthologs to the glucosinolate transporters GTR1 and GTR2 in the pennycress transcriptome. These predicted peptides have significant homology to the Arabidopsis GLUCOSINOLATE TRANSPORTER 1-2 peptides (GTR1, *At3G47960*; GTR2, *At5G62680*).

This comparison of assembly coverage is at least qualitatively indicative of expression level differences in the total RNA library. Although directly comparing the non-normalized statistic of mean coverage across transcripts for quantification is inappropriate, we observed many high-coverage transcripts related to glucosinolate metabolism. Interestingly, we observed that, among the 100 transcripts with the highest mean coverage, six were similar to β-glucosidase (two transcripts), myrosinases (three transcripts) and myrosinase-binding protein (one transcript). The remaining 94 transcripts in this group may be considered ‘housekeeping’ genes. Predictably, most of these transcripts are involved in photosynthetic processes. It remains unknown whether the high levels of glucosinolates and glucosinolate by-products in pennycress are simply due to high expression of these myrosinates and/or specifier proteins, unique activity, unique hormonal regulation of activity or expression, or some combination of these.

## Discussion

### Comparative transcriptomics of pennycress and Arabidopsis

We have sequenced, assembled and annotated the pennycress transcriptome. The draft transcriptome consists of 33 873 unique sequences, of which 27 442 were annotated using the Blast2GO pipeline. Of these transcripts, 35% were most similar to an *A. thaliana* gene, and 74% had top hits in the Brassicaceae, indicating a high level of sequence conservation across the family. blast comparisons between pennycress and five other sequenced Brassicaceae species showed that our pennycress transcriptome has good coverage of homologous sequences. These analyzes are consistent with previous phylogenetic findings indicating that pennycress is more closely related to *T. halophila* than to *Brassica* species (Franzke *et al*., 2011).

The total transcriptome assembly length was over 42 Mbp. The pennycress genome (2*n* = 14) is approximately 539 Mbp (Hume *et al*., 1995; Johnston, 2005). In comparison, the Arabidopsis genome (2*n* = 10) is estimated to be 125 Mbp (Kaul *et al*., 2000), with the latest genome annotation release (TAIR10) containing 33 602 genomic features, including 27 416 protein-coding genes. *Brassica rapa* has 41 174 protein-coding genes, with mean transcript/coding lengths of 2015/1172 bp (Wang *et al*., 2011b). The number of genes identified here in pennycress, together with the estimated genome size, matches similar observations on total gene number in the Arabidopsis and *B. rapa* genomes.

### Characterization of pennycress glucosinolate metabolism and translocation

Many plants in the order Brassicales produce high levels of glucosinolates and glucosinolate hydrolysis products, which are thought to provide a defensive function (Bones and Rossiter, 1996). Glucosinolates are one of the most highly characterized secondary metabolites in Arabidopsis (Wittstock and Burow, 2010). Myrosinases, also known as thioglucoside glucohydrolases, hydrolyze the glucosinolate, forming an intermediate aglycone. The aglycone is either spontaneously rearranged to form isothiocyanates, or converted to a simple nitrile, epithionitrile or thiocyanate by specifier proteins. The characteristic ‘garlic-like’ odor of pennycress has led researchers to investigate the levels of glucosinolates and glucosinolate by-products in pennycress (Warwick *et al*., 2002; Kuchernig *et al*., 2011). This has led to another common name for this species: ‘stinkweed’. A single thiocyanate-forming protein has previously been identified and characterized in pennycress (Kuchernig *et al*., 2011). Pennycress seed has also been investigated for its biofumigant properties – probably due to the high levels of glucosinolates in the seeds (Vaughn *et al*., 2005). After oil is pressed from pennycress seed, the remaining press cake has high levels of protein (25%), which has the potential to serve as an animal feed supplement or for use in industrial products (Selling *et al*., 2013). However, the high levels of glucosinolates, which may be toxic to animals, prohibit such use (Best and Mcintyre, 1975; Warwick *et al*., 2002; Vaughn *et al*., 2005). Previous work in Arabidopsis identified key glucosinolate transporters that are responsible for translocating glucosinolates (Nour-Eldin *et al*., 2012). The Arabidopsis double mutant *gtr1 gtr2* showed significantly reduced levels of glucosinolates in seed. We predict that loss-of-function mutations in the pennycress GTR-like genes identified here would cause a reduction in seed glucosinolate levels.

### Genetics of flowering time in winter annual pennycress

The genetic mechanisms controlling the transition from vegetative to reproductive growth have been widely studied in Arabidopsis and other plant species (Simpson, 2002; Amasino, 2005; Kim *et al*., 2009). In many species adapted to winter climates, a period of cold provided by over-wintering is required to make plants competent to flower, a process that is known as vernalization. In many crucifer species, there is natural variation in populations adapted to different climates. Much of this variation is attributed to the complex interaction of FRIGIDA (FRI), the FRIGIDA protein complex and FLOWERING LOCUS C (FLC), which provide the main response to vernalization (Choi *et al*., 2011). The period of vernalization provided by winter epigenetically represses FLC expression (Sheldon *et al*., 2000; Michaels and Amasino, 2001). This removes the transcriptional repression by FLC on FLOWERING LOCUS T (FT), a main integrator of environmental cues promoting flowering. ‘Fast-cycling’ lines of Arabidopsis contain a loss-of-function mutation in FRI (Johanson *et al*., 2000; Gazzani, 2003).

Variation of FRI and FLC orthologs in *B. rapa* (Schranz *et al*., 2002; Yuan *et al*., 2009), *B. oleracea* (Irwin *et al*., 2012) and *B. napus* (Tadege *et al*., 2001; Wang *et al*., 2011a) is associated with vernalization and flowering. Both ‘early’ and ‘late’ flowering lines of pennycress have been reported (Best and Mcintyre, 1976). Much like the fast cycling lines of Arabidopsis, the ‘early’ pennycress lines flower without a period of vernalization, exhibiting a spring annual habit. The late-flowering lines grow for a period of time in the fall (autumn) as a vegetative rosette, but do not flower until the spring. The genetic differences between winter and spring annual pennycress lines were determined to be caused by a single dominant allele (Mcintyre and Best, 1978). We predict that the natural variation between spring and winter lines is due to mutations in FRI or FLC-like genes. In order for pennycress to be easily used as a winter cover crop in various climates, precise control of spring flowering time is required. Perturbations of the flowering time pathway in cultivated species through breeding and genetic modification have served as an important tool for controlling flowering time (Jung and Müller, 2009). Our identification of likely orthologous genes responsible for controlling flowering time will be a useful tool for making rapid improvements in the pennycress germplasm.

### *Considerations regarding* de novo *transcriptome assembly*

Varying *de novo* assembly parameters using short-read data has been shown to enable assembly of unique transcripts corresponding to real genes (Zhao *et al*., 2011). In this study, we chose a single assembly based on the high quality assembly statistics and the high number of transcripts with significant similarity to Arabidopsis peptides. The finding of a high number of potentially orthologous sequences in the pennycress and its relatives provides a validation of the pennycress assembly. However, different assembly programs and parameters affect the assembly of transcripts expressed at both high and low levels (Zhao *et al*., 2011; Gongora-Castillo and Buell, 2013). For example, *de novo* assemblies created with large word sizes poorly assemble lowly expressed genes (Gruenheit *et al*., 2012). Thus, it is not expected that any one assembly will truly represent the complete biological transcriptome. This was highlighted in the current analysis between pennycress and Arabidopsis. We predicted that a LEAFY-like ortholog should be represented in our RNA pools, but it was not found in the final assembly. Assemblies with smaller word sizes (24, 30, and 46) did assemble LEAFY-like transcripts (see transcript sequences in Data S1). These transcripts had low coverage (7× mean) with few mapped reads. Combined with the high number of reads used to create our final assembly (over 200 million), this indicates that the pennycress LEAFY ortholog was expressed at low levels in our sample and is probably not included in the final assembly due to the larger word size. In our optimization, smaller word sizes also resulted in assembly of some obviously mis-assembled transcripts in which multiple transcripts from unlinked genes were joined together. These results further support the requirement for full characterization of the potential changes caused by various *de novo* assembly parameters.

### Future perspectives

We have identified pennycress transcripts that are probably responsible for controlling key agronomic traits such as seed glucosinolate levels and flowering time, which are primary targets for future research in order to improve the pennycress germplasm. It should be straightforward to make improvements using reverse genetic approaches to identify inactive or altered alleles by using well-established TILLING protocols (McCallum *et al*., 2000; Kurowska *et al*., 2011). Our ongoing sequencing of the pennycress genome will enable rapid screening of TILLING populations through next-generation sequencing. In addition, the ability to make improvements using transgenic approaches to modify gene expression should soon be possible as we have found that pennycress is relatively easy to regenerate *in vitro* (Matthew Krause, Kevin M. Dorn, M. David Marks, unpublished observation). Pennycress has tremendous agronomic potential as a winter cover and new source of oilseeds. A recent report by the Massachusetts Institute of Technology Joint Program on Science and Policy of Global Change indicates that pennycress could be grown on over 40 million acres each year, yielding up to 6 billion gallons of oil that may be converted to biodiesel (Moser *et al*., 2009a; Winchester *et al*., 2013). This represents approximately 15% of the 40 billion gallons of diesel consumed annually in the USA. The recent advances in ‘omics-based’ technologies will allow for the use of the resources developed here to make rapid improvements to the pennycress germplasm.

## Experimental Procedures

### Plant growth conditions and RNA extraction

Seed from a small natural population of *T. arvense* L. was collected near Coates, MN. Seeds were planted in moist Berger BM2 germination mix (Berger Inc., http://www.berger.ca), stratified for 7 days at 4°C, and then placed in a 21°C growth chamber. Individual seedlings were transferred to 4-inch pots after 2 weeks, and were grown under banks of AgroMax 6400K T5 fluorescent lights (HTGSupply, http://www.htgsupply.com) with a 16 h/8 h day/night cycle at 98 micromoles/m^2^/s PAR. To initiate flowering, 6-week-old plants with established rosettes were covered and transferred to a 4°C cold room for 14–29 days in the dark. After vernalization, plants were transfered back to the growth chamber and grown under 400 W metal halide bulbs (Philips, http://www.usa.lighting.philips.com) at 50 micromoles/m^2^/s PAR. Roots, hypocotyls, cotyledons and young leaves were obtained by planting sterilized seed on 1× Murashige and Skoog medium with 0.8% agar. Seed was stratified at 4°C for 3 days, and then grown for 7 days in constant light under T12 fluorescent bulbs (Philips) at 42 micromoles/m^2^/s PAR.

RNA was extracted from (i) roots from 12 seedlings grown on MS plates, (ii) hypocotyls, cotyledons, young meristems and first leaves from 12 seedlings grown on MS plates, (iii) four new leaves from each of two 120-day-old unvernalized plants, (iv) aerial leaves and stems from 128-day-old flowering plants, and (v) flowers and seed pods from 128-day-old flowering plants. RNA was purified using an RNeasy plant mini kit (Qiagen, http://www.qiagen.com) according to the manufacturer's instructions. Following the initial total RNA extraction, samples were treated with Ambion TURBO DNase (Life Technologies, http://www.lifetechnologies.com) according to the manufacturer's instructions, immediately followed by the RNA clean-up procedure from the Qiagen RNeasy kit.

### High-throughput RNA sequencing and *de novo* assembly

A pooled sample containing equal amounts of purified total RNA from each of the five tissue samples was submitted to the University of Minnesota Biomedical Genomics Center for sequencing. RNA was subjected to quality control using the Invitrogen RiboGreen RNA assay (Life Technologies), and RNA integrity was analyzed by capillary electrophoresis on an Agilent BioAnalyzer 2100 (Agilent Technologies, http://www.agilent.com). Polyadenylated RNA was selected using oligo(dT) purification and reverse-transcribed to cDNA. cDNA was fragmented, blunt-ended, and ligated to the Illumina TruSeq Adaptor Index 3 (Illumina Inc., http://www.illumina.com). The library was size-selected for an insert size of 200 bp, and quantified using the Invitrogen PicoGreen dsDNA assay (Life Technologies). The pooled RNA sample was sequenced using the Illumina HiSeq 2000 platform using 100 bp, paired-end reads, producing 374 million reads above Q30. Read pairs had a mean insert size of 200 bp. Duplicate reads were removed, and the first 10 nucleotides were trimmed from the 5′ end of each read using the tools in the CLC Genomics Workbench 5.5 (CLC Bio, http://www.clcbio.com). The additional trimming parameters were: removal of low-quality sequence limit = 0.05; removal of ambiguous nucleotides, maximum two nucleotides allowed; removal of terminal nucleotides, 10 nucleotides from the 5′ end; removal of Illumina TruSeq Indexed Adaptor 3 and Universal Adapter sequences.

Reads were *de novo* assembled into contigs using the CLC Genomics Workbench 5.5 *de novo* assembly tool. A series of independent assemblies were performed to analyze the effects of varying the *de novo* assembly parameters. Assemblies were performed using varying word size (18, 24, 30, 36, 40, 46, 52, 58 and 64), and with length fractions (match length) of 0.7 and 0.95. An additional 23 assemblies were performed using values outside these parameters, with a total of 41 assemblies performed. The remaining assembly parameters were: auto bubble size, yes; minimum contig length, 300 bp; perform scaffolding, yes; mismatch cost, 3; insertion cost, 3; deletion cost, 3; update contigs, yes. Functional annotations and gene ontologies were assigned to each assembled contig from the final assembly using Blast2GO with the following parameters: BLASTx against the NCBI non-redundant protein database, BLAST E-value = 0.001, and reporting the top 20 hits. Comparative blast searches against Arabidopsis were performed using the CLC Genomics Workbench blast function, using sequences obtained from the TAIR10 release of the Arabidopsis transcriptome and proteome (http://www.arabidopsis.org) (Lamesch *et al*., 2012). Sequences for *Arabidopsis lyrata* (Hu *et al*., 2011), *Capsella rubella* (Slotte *et al*., 2013), *B. rapa* (Wang *et al*., 2011b) and *T. halophila* were obtained from Phytozome v9.1 (http://www.phytozome.net). Further statistical analysis and figures were prepared using r (R Development Core Team, 2008). The final assembly described here has been submitted to DDBJ/EMBL/GenBank under the accession GAKE01000000. The complete, annotated FASTA file is available at http://www.cbs.umn.edu/lab/marks/pennycress/transcriptome.

## SUPPORTING INFORMATION

**Figure S1.** Most highly represented GO terms in the pennycress transcriptome annotation.

**Figure S2.** Global comparison of the Arabidopsis and pennycress transcriptomes.

**Figure S3.** Reconstruction of the flowering time pathway in pennycress.

**Table S1.** Effects of *de novo* assembly parameters on assembly statistics.

**Table S2.** Transcript annotations, GO terms and top blast hits to Arabidopsis.

**Table S3.** BLASTx results of pennycress transcriptome against five Brassicaceae species,

**Table S4.** tBLASTn results of five global blast comparisons of five Brassicaceae species to the pennycress transcriptome assembly.

**Data S1.** Predicted peptide sequences for candidate orthologs involved in flowering and glucosinolate metabolism.
