# Supplementary material for: De novo assembly of the pennycress (Thlaspi arvense) transcriptome provides tools for the development of a winter cover crop and biodiesel feedstock
Source: Plant J. 2013 Jun 20;75(6):1028–38. doi: 10.1111/tpj.12267 (PMC3824206; doi:10.1111/tpj.12267)
Supplement: Supplementary file 1 [file tpj0075-1028-SD1.pdf]

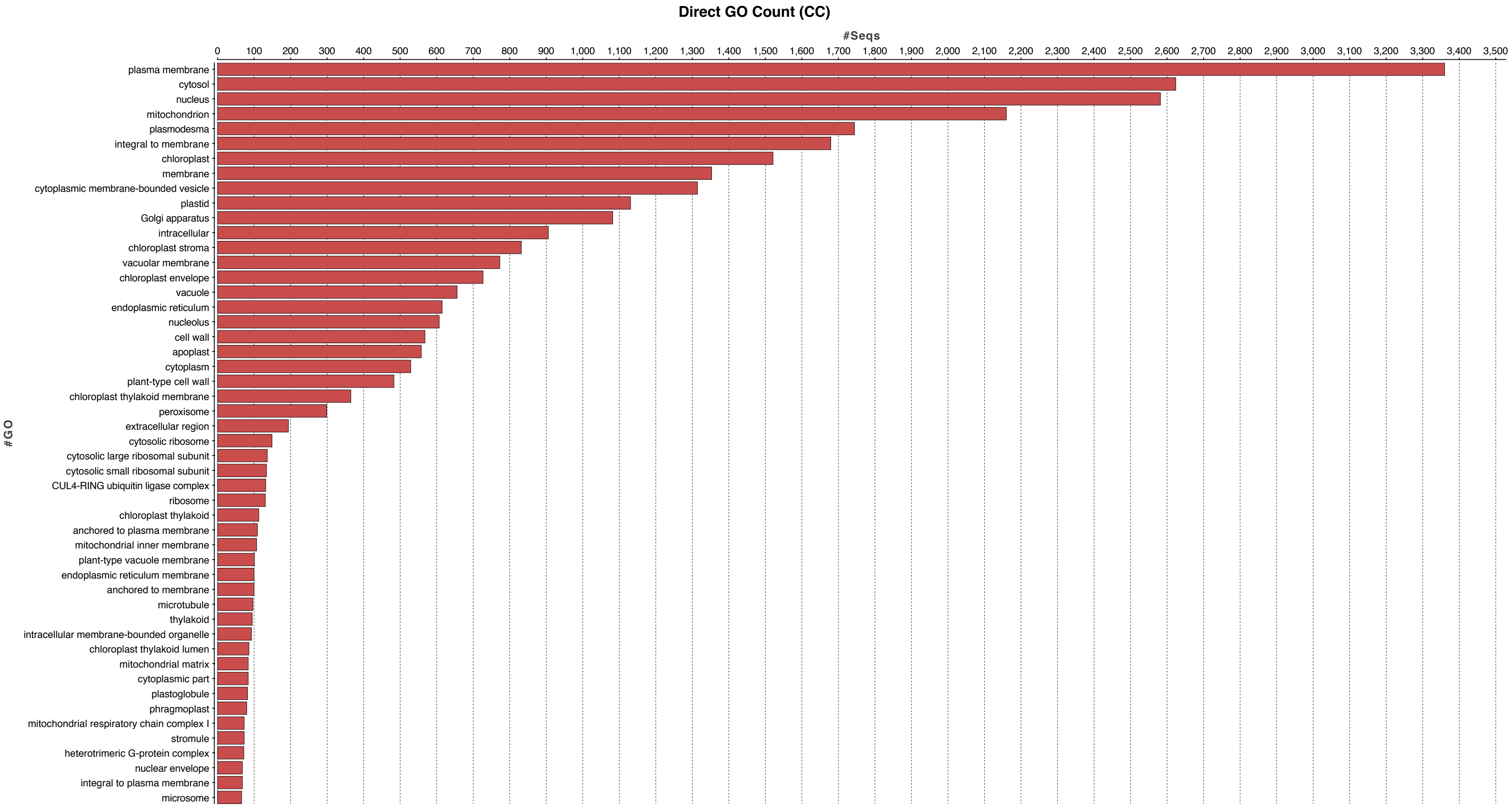

Supporting Figure S1 – Most highly represented GO terms in the pennycress transcriptome annotation
GO terms were assigned to each transcript using the Blast2GO pipeline. The most highly represented biological process, cellular component, and molecular function terms are represented.

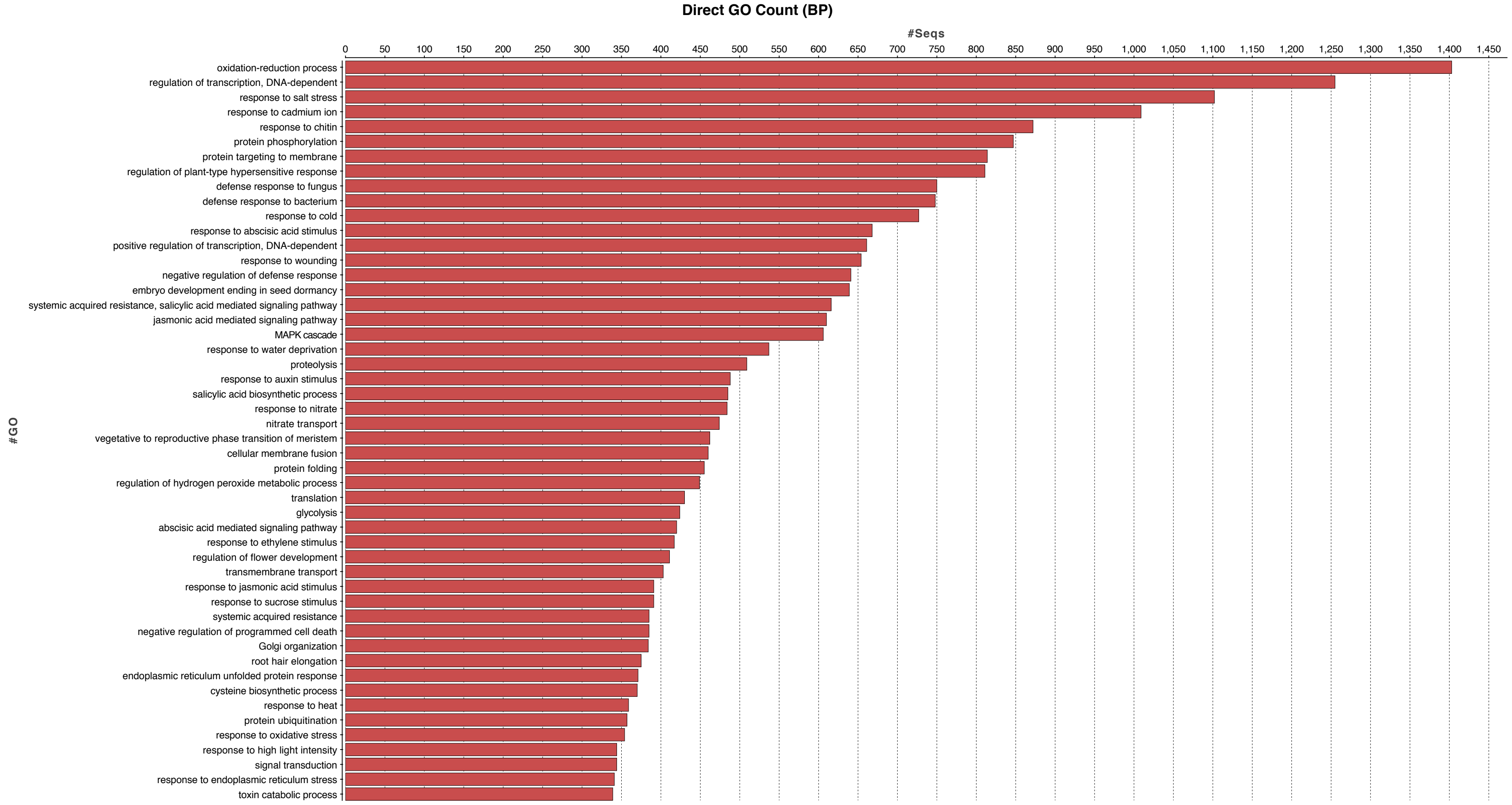
Direct GO Count (BP)
#Seqs
0
50
100
150
200
250
300
350
400
450
500
550
600
650
700
750
800
850
900
950
1,000
1,050
1,100
1,150
1,200
1,250
1,300
1,350
1,400
1,450
#GO
oxidation-reduction process
regulation of transcription, DNA-dependent
response to salt stress
response to cadmium ion
response to chitin
protein phosphorylation
protein targeting to membrane
regulation of plant-type hypersensitive response
defense response to fungus
defense response to bacterium
response to cold
response to abscisic acid stimulus
positive regulation of transcription, DNA-dependent
response to wounding
negative regulation of defense response
embryo development ending in seed dormancy
systemic acquired resistance, salicylic acid mediated signaling pathway
jasmonic acid mediated signaling pathway
MAPK cascade
response to water deprivation
proteolysis
response to auxin stimulus
salicylic acid biosynthetic process
response to nitrate
nitrate transport
vegetative to reproductive phase transition of meristem
cellular membrane fusion
protein folding
regulation of hydrogen peroxide metabolic process
translation
glycolysis
abscisic acid mediated signaling pathway
response to ethylene stimulus
regulation of flower development
transmembrane transport
response to jasmonic acid stimulus
response to sucrose stimulus
systemic acquired resistance
negative regulation of programmed cell death
Golgi organization
root hair elongation
endoplasmic reticulum unfolded protein response
cysteine biosynthetic process
response to heat
protein ubiquitination
response to oxidative stress
response to high light intensity
signal transduction
response to endoplasmic reticulum stress
toxin catabolic process

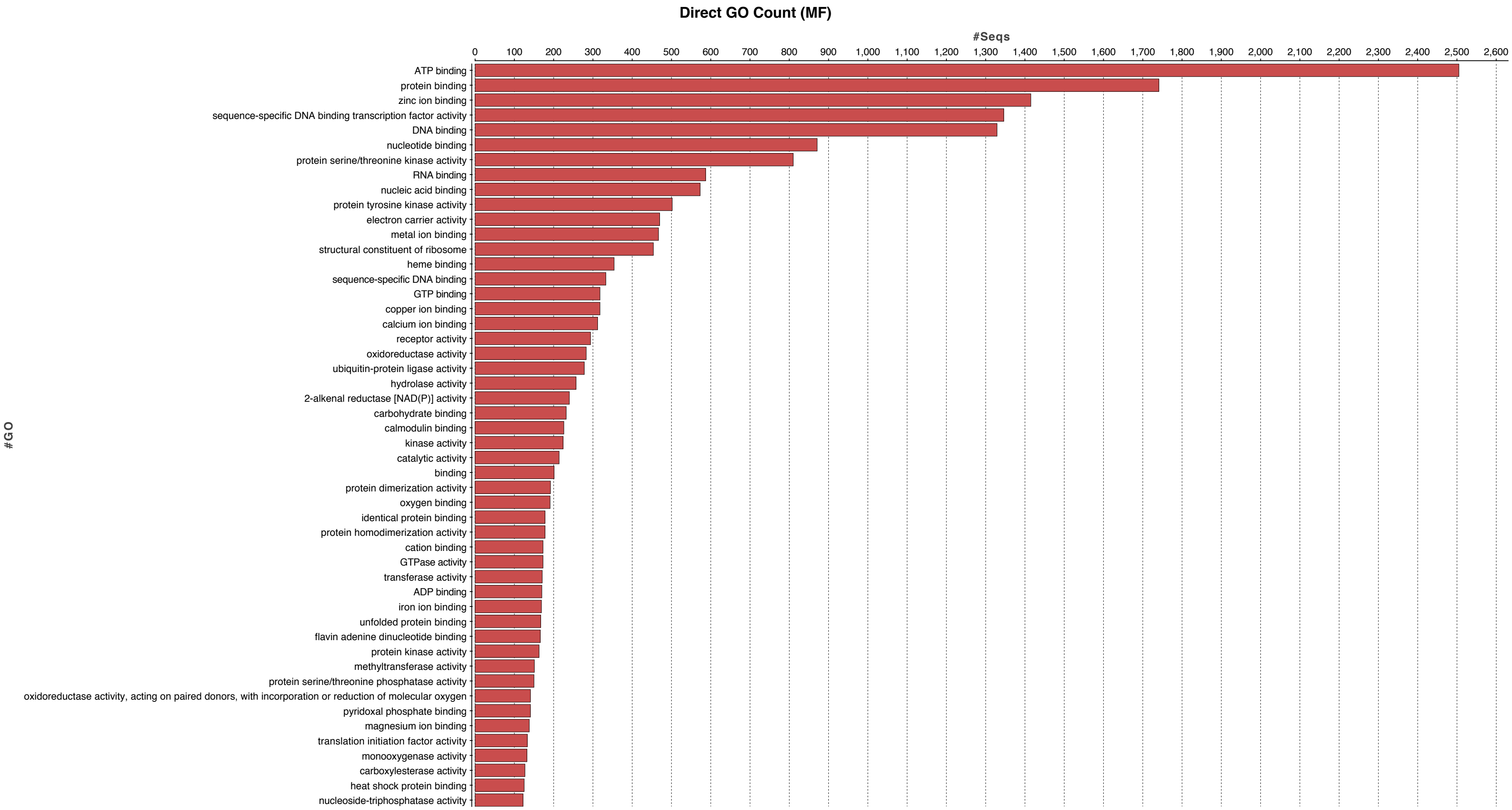

Direct GO Count (MF)
#Seqs
0
100
200
300
400
500
600
700
800
900
1,000
1,100
1,200
1,300
1,400
1,500
1,600
1,700
1,800
1,900
2,000
2,100
2,200
2,300
2,400
2,500
2,600
#GO
ATP binding
protein binding
zinc ion binding
sequence-specific DNA binding transcription factor activity
DNA binding
nucleotide binding
protein serine/threonine kinase activity
RNA binding
nucleic acid binding
protein tyrosine kinase activity
electron carrier activity
metal ion binding
structural constituent of ribosome
heme binding
sequence-specific DNA binding
GTP binding
copper ion binding
calcium ion binding
receptor activity
oxidoreductase activity
ubiquitin-protein ligase activity
hydrolase activity
2-alkenal reductase [NAD(P)] activity
carbohydrate binding
calmodulin binding
kinase activity
catalytic activity
binding
protein dimerization activity
oxygen binding
identical protein binding
protein homodimerization activity
cation binding
GTPase activity
transferase activity
ADP binding
iron ion binding
unfolded protein binding
flavin adenine dinucleotide binding
protein kinase activity
methyltransferase activity
protein serine/threonine phosphatase activity
oxidoreductase activity, acting on paired donors, with incorporation or reduction of molecular oxygen
pyridoxal phosphate binding
magnesium ion binding
translation initiation factor activity
monooxygenase activity
carboxylesterase activity
heat shock protein binding
nucleoside-triphosphatase activity
