# Supplementary material for: De novo assembly of the pennycress (Thlaspi arvense) transcriptome provides tools for the development of a winter cover crop and biodiesel feedstock
Source: Plant J. 2013 Jun 20;75(6):1028–38. doi: 10.1111/tpj.12267 (PMC3824206; doi:10.1111/tpj.12267)
Supplement: Supplementary file 2 [file tpj0075-1028-SD2.pdf]

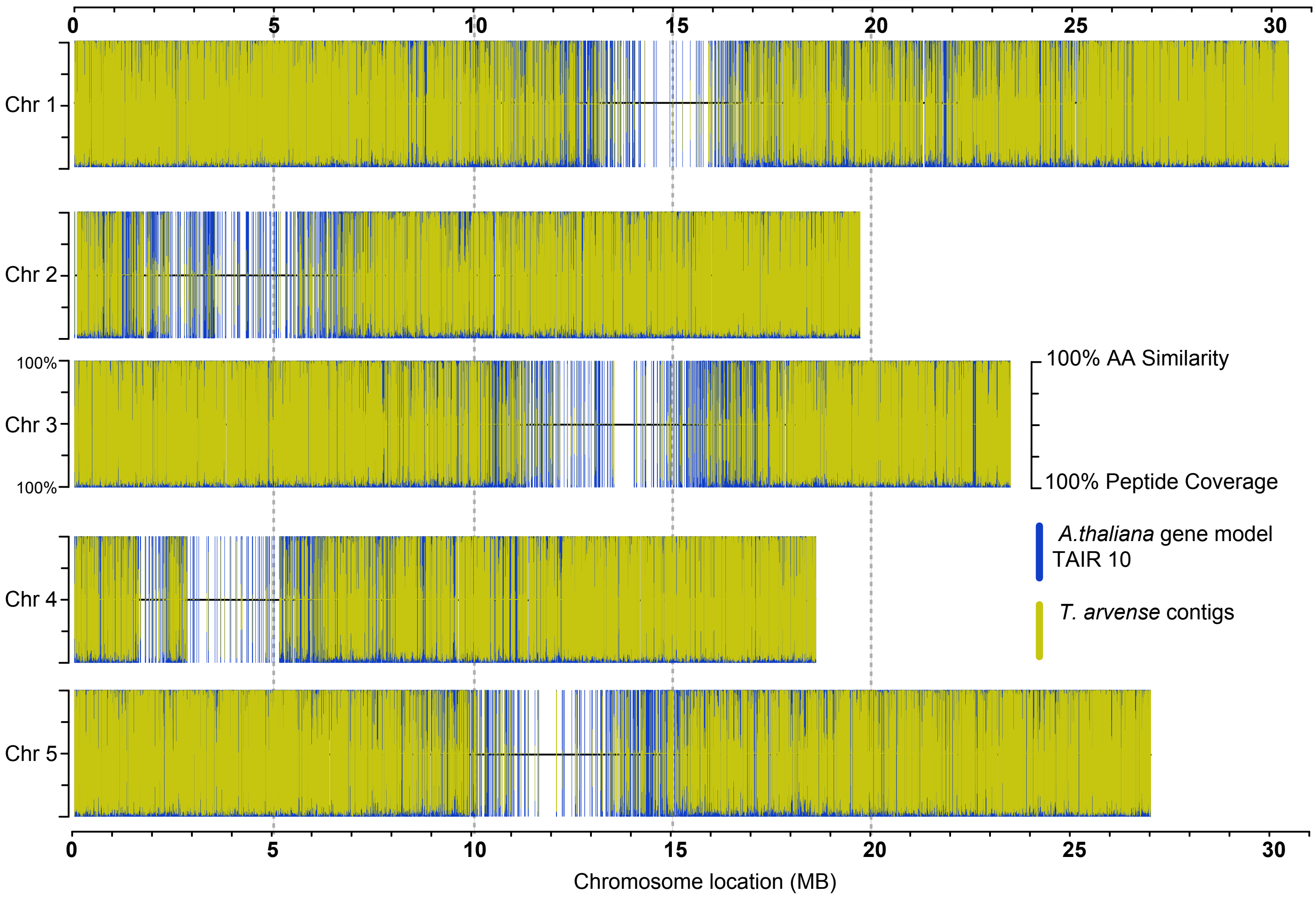

Supporting Figure S2 - Global comparison of the *Arabidopsis* and pennycress transcriptomes

Global representation of the *Arabidopsis* transcriptome reconstructed using pennycress transcripts. Each of the five *Arabidopsis* chromosomes (Chr 1-5) is shown with each gene model relative to chromosomal position with blue bars. The percent of sequence similarity is shown in the positive y-axis, and percent of the *Arabidopsis* peptide covered with this similarity in the negative y-axis (yellow bars). White regions represent non-genic regions. The similarity and coverage of the most similar pennycress transcript is shown in yellow for each *Arabidopsis* gene.
