# Supplementary material for: De novo assembly of the pennycress (Thlaspi arvense) transcriptome provides tools for the development of a winter cover crop and biodiesel feedstock
Source: Plant J. 2013 Jun 20;75(6):1028–38. doi: 10.1111/tpj.12267 (PMC3824206; doi:10.1111/tpj.12267)
Supplement: Supplementary file 3 [file tpj0075-1028-SD3.pdf]

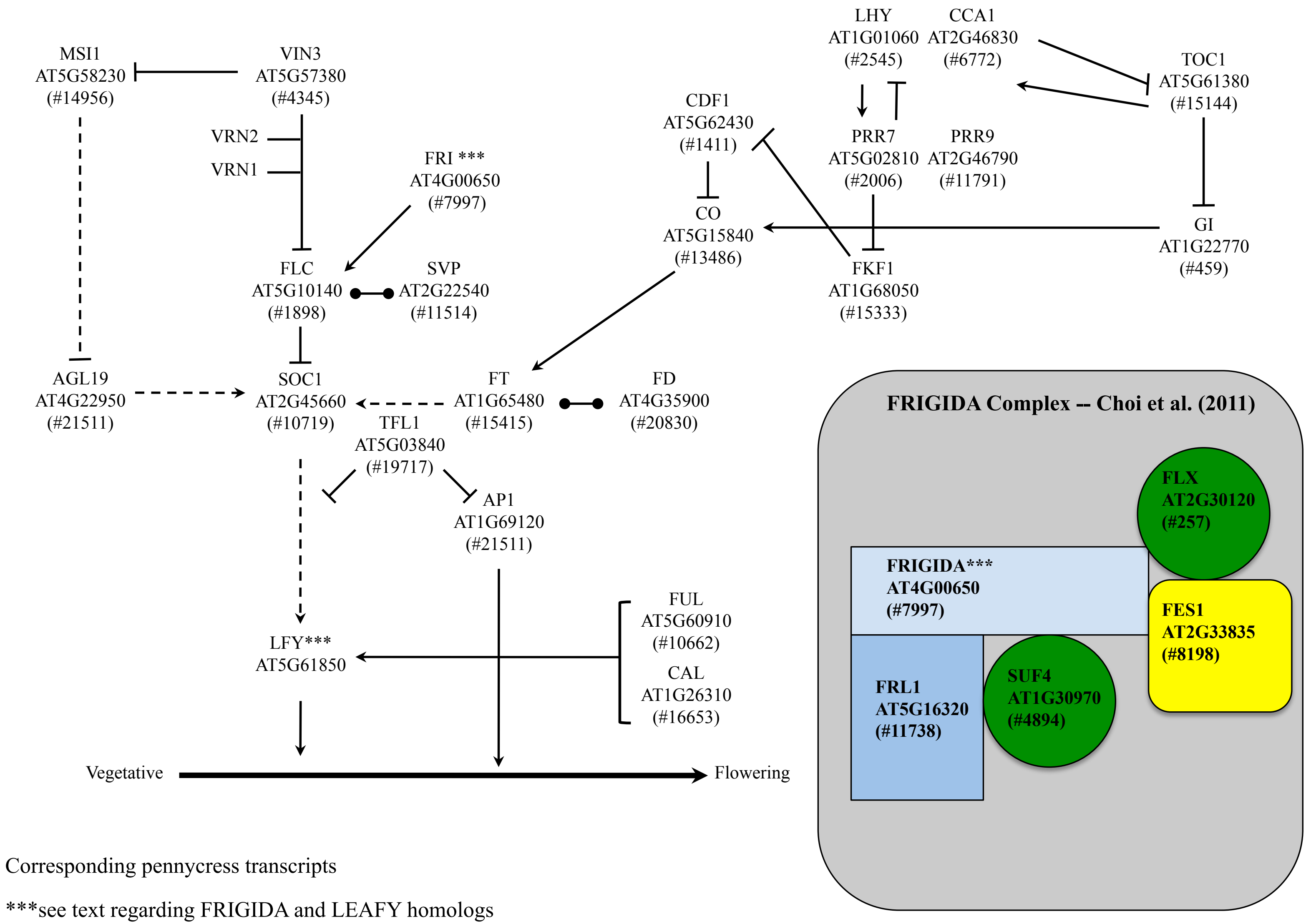

Corresponding pennycress transcripts

***see text regarding FRIGIDA and LEAFY homologs

Supporting Figure S3 – Reconstruction of the flowering time pathway in pennycress
A model for the pathway controlling flowering time in pennycress adapted from Jung and Müller (2009).
